# Optimization of Enzyme−Assisted Aqueous Extraction of Polysaccharide from *Acanthopanax senticosus* and Comparison of Physicochemical Properties and Bioactivities of Polysaccharides with Different Molecular Weights

**DOI:** 10.3390/molecules28186585

**Published:** 2023-09-12

**Authors:** Xueyan Wang, Yuanyuan Su, Jianqing Su, Jiaojiao Xue, Rui Zhang, Xiaoli Li, Ying Li, Yi Ding, Xiuling Chu

**Affiliations:** 1 College of Agronomy and Agricultural Engineering, Liaocheng University, Liaocheng 252000, China; wangxueyan202203@163.com (X.W.); x15065441211@163.com (J.X.); 17863709708@163.com (R.Z.); lxl15006995983@163.com (X.L.); ly15963373832@163.com (Y.L.); djy15373023047@163.com (Y.D.); 2 Chinese Materia Medica College, Tianjin University of Traditional Chinese Medicine, Tianjin 301617, China; 13894200757@163.com

**Keywords:** *Acanthopanax senticosus* polysaccharide, enzyme−assisted aqueous extraction, different molecular weights

## Abstract

To obtain the optimal process for the enzyme−assisted aqueous extraction of polysaccharides from *Acanthopanax senticosus*, and study the physicochemical properties of polysaccharides of different molecular weights, the extraction of *Acanthopanax polysaccharides* was optimized using the BBD response surface test. The polysaccharides with different molecular weights were obtained by ethanol−graded precipitation at 40%, 60%, and 80%, which were presented as ASPS40, ASPS60, and ASPS80. The polysaccharides were analyzed by HPGPC, ion chromatography, FT−IR, UV, SEM, TGA, XRD, Congo red, and I_2_−KI tests. The antioxidant assay was used to evaluate their antioxidant properties in vitro. The findings demonstrated that the recovery rate of *Acanthopanax polysaccharide* was 10.53 ± 0.682%, which is about 2.5 times greater compared to the conventional method of hot water extraction. Based on FT−IR, TGA, polysaccharides with different molecular weights did not differ in their structure or thermal stability. The XRD suggests that the internal structure of ASPSs is amorphous. Congo red and I_2_−KI showed that all three polysaccharides had triple helix structures with longer branched chains and more side chains. Furthermore, the antioxidant results showed the antioxidant activity of polysaccharides is not only related to the molecular weight size but also can be related to its composition and structure. These studies developed a green, and scalable method to produce polysaccharides from *Acanthopanax senticosus* and evaluated the properties of *Acanthopanax* polysaccharides of different molecular weights.

## 1. Introduction

*Acanthopanax senticosus* (AS) is a shrub plant belonging to *Acanthaceae*, mainly distributed in Russia, Japan, Heilongjiang, and Jilin of China. AS contains multiple nutrients and components as a medicinal and food homologous plant. AS has a variety of pharmacological activities, such as immunomodulatory [1], antioxidant [2], anti−inflammatory [3], and anti−tumor [4]. Based on the numerous pharmacological functions of polysaccharides, it has become a hot topic to study the extraction methods of polysaccharides to enhance their content without destroying the active ingredients. *Acanthopanax* polysaccharides are extracted mainly through hot water extraction, acid−base extraction, ultrasonic−assisted extraction, and enzyme−assisted extraction. However, each method has its shortcomings. For instance, hot water extraction uses the principle of “similar compatibility” to extract polysaccharides while other water−soluble components are also extracted, and high extraction temperature results in low purity of polysaccharides, long time consumption, and low extraction rate. The acid−base extraction method has the disadvantage of easily destroying the structure of polysaccharides and reducing the pharmacological activity of polysaccharides [5]. Although the ultrasonic−assisted extraction method can destroy the cell wall of plants and improve the extraction rate, it has the problems of slow and time−consuming temperature rise and high energy consumption [6]. Enzyme−assisted extraction technology uses the property of enzymes to break down plant tissues and accelerate the release of polysaccharides. It is an effective extraction method that is gentle, specific, quick, high rate, environmentally friendly, and maintains the biological activity of polysaccharides [7]. Various enzymes are widely used to extract bioactive components, such as cellulase, pectinase, hemicellulase, and amylase. Cellulases are enzymes that break down cellulose into glucose, cellobiose, and higher glucose polymers; Pectinases can break down pectin compounds [8]; Hemicellulases can break down hemicellulose in plant cell walls and accelerate polysaccharide solubilization [9]. Several studies have demonstrated the advantages of enzymes in extracting plant polysaccharides. Gao et al. [10] extracted *E. sibiricumbulb* polysaccharides with amylase, and the yield reached 59.71 ± 2.72% after optimization of extraction conditions, which was much higher than that of hot water extraction (37.25 ± 0.17%). Shi et al. [11] extracted *Dendrobium* polysaccharides with the aid of a combination of cellulase and pectinase, resulting in a much higher extraction rate. Wang et al. [12] extracted *pumpkin* seed polysaccharides with cellulase and compared the yield with hot water extraction and ultrasonic extraction and concluded that the enzyme extraction (3.22% ± 0.04%) gave a higher yield than hot water extraction (2.18%) and ultrasonic extraction (2.29%). The molecular weights of the polysaccharides obtained by the three extraction methods were different, indicating that the extraction methods affect the molecular weight of polysaccharides.

The bioactivity of polysaccharides is influenced by molecular weight [13]. Screening the molecular weight range for better activity is conducive to improving the bioavailability of polysaccharides and developing polysaccharides with better action. Ethanol graded precipitation method is often used to separate polysaccharides of different molecular weight sizes. The precipitation using different ethanol concentrations can yield polysaccharide fractions of various molecular weights, which have other properties and can be used in different pathways [14]. Therefore, ethanol−graded precipitation is widely used. In studying *dandelion* polysaccharides, one researcher used ethanol graded precipitation method to obtain two polysaccharide fractions of DRP−2b and DRP−3a with sizes of 31.8 kDa and 6.72 kDa, respectively, and the antioxidant capacity of DRP−3a was higher than that of DRP−2b at a concentration of 1 mg/mL, and the clearance of DPPH by DRP−2b and DRP−3a was 28.82 ± 2.45% and 71.58 ± 3.11%, respectively [15]. Long investigated the binding ability of different fractions of hot water−extracted *Caulerpa lentillifera* polysaccharides to bile acids after graded precipitation by ethanol [16]. The findings demonstrated that the smaller molecular weights of WCLP−55 and WCLP−75 had a much better bile acid binding ability than the other fractions [17]. In their study of the hypoglycemic effect of *senna* polysaccharides precipitated from different ethanol concentrations (20%, 40%, 60%, and 80%), they found that HP80 with the minimum molecular weight of molecular weight showed the most potent inhibitory activity against α−amylase and α−glucosidase when they investigated the hypoglycemic effect of senna polysaccharides precipitated by different ethanol concentrations. Gao et al. [18] isolated *lychee* polysaccharides using four different methods and discovered that those with the highest molecular weight had the most antioxidant action. In recent years, more and more researchers have obtained polysaccharides of different molecular weight sizes by graded precipitation of ethanol, intending to develop a highly bioavailable polysaccharide.

At present, most of the studies on *Acanthopanax* polysaccharides have focused on their pharmacological effects. Few studies have been reported on the extraction process and physicochemical properties of *Acanthopanax* polysaccharides, which are the key factors for their practical application. Therefore, it is worthwhile to conduct research into the *Acanthopanax* polysaccharide extraction procedure and its physicochemical characteristics. In this paper, three enzymes, cellulase, hemicellulase, and pectinase, were selected for the extraction of *Acanthopanax* polysaccharides by the complex enzyme−assisted hot water method. The response surface was used to optimize the extraction procedure. Ethanol−graded precipitation was used to separate polysaccharides with different molecular weights. Physicochemical properties of different molecular weights of polysaccharides were compared by HPGPC, ion chromatography, FT−IR, UV, SEM, TGA, XRD, Congo red, and I_2_−KI. It provides some technical support for the clinical application of *Acanthopanax* polysaccharide.

## 2. Results

### 2.1. Compound Enzyme Ratio Optimization

#### 2.1.1. Enzyme Dosage Screening

(1) The effect of cellulase dosage on polysaccharide yield

When cellulase was added from 12.5 U/g to 800 U/g, the yield of *Acanthopanax* polysaccharide showed a significant trend. It may be because cellulase can loosen and break down cell walls and reduce their mass transfer resistance, allowing polysaccharides to precipitate easily [19]. However, the difference between the polysaccharide yield at 800 U/g and 400 U/g was insignificant (*p* < 0.05), and 400 U/g was selected as the best amount of cellulase addition in combination with cost and other factors.

(2) The effect of pectinase dosage on polysaccharide yield

The polysaccharide yields increased faster when the pectinase addition was increased from 12.5 U/g to 50 U/g. It may be because, as the amount of enzyme increases, the contact area between the enzyme and the substrate increases, and the reaction is accelerated, making the polysaccharide yield rise rapidly. The polysaccharide yield showed a slowly increasing trend when the addition amount was increased from 50 U/g to 100 U/g. The extraction rate reached a maximum of 8.22% at 100 U/g, and then the polysaccharide extraction rate started to decrease when the pectinase addition was increased again. This phenomenon may be because when too much enzyme is added, more reactants are produced, inhibiting the reaction rate and decreasing the polysaccharide yield [20].

(3) The effect of hemicellulase dosage on polysaccharide yield

When the hemicellulase addition was increased from 25 U/g to 200 U/g, the yield of *Acanthopanax* polysaccharide showed an increasing trend, and the extraction rate reached the maximum value of 7.46% at the additional level of 200 U/g. The polysaccharide yield decreased when the amount of hemicellulase was increased again, and the reason for the change here was consistent with the effect of pectinase on the polysaccharide yield of *A. senticosus*.

#### 2.1.2. Orthogonal Optimization of Complex Enzyme Ratios

**Figure 1:**
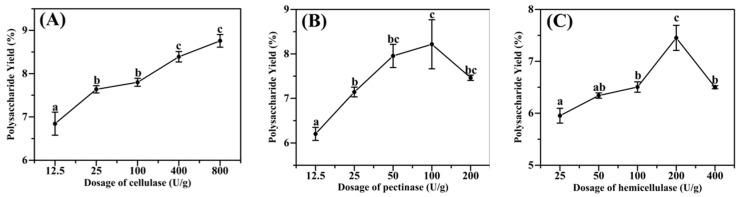
The effect of each enzyme on the yield of polysaccharides from *A. senticosus*: (**A**) the amount of cellulase; (**B**) the amount of pectinase; (**C**) the amount of hemicellulase. Note: a, b, and c: represent the significance of the difference within the graph. Containing the same letter means the difference is insignificant (*p* > 0.05).

Based on the above single factors, the optimal amounts of three single enzymes (cellulase, pectinase, and hemicellulase) can be derived as 400 U, 100 U, and 200 U. Using a three−factor, three−level orthogonal experiment, the conditions for the optimal ratio of complex enzymes were optimized. The R−values of 0.62 > 0.30 > 0.25 indicated that cellulase had the greatest impact on the extraction rate, followed by pectinase and hemicellulase. The highest polysaccharide extraction of 8.76% by cellulase can also be seen in the single enzyme extraction test above (Figure 1A). From Table 1, it can be seen that the highest polysaccharide yield of 9.07% was obtained in group 3, which was higher than that of any of the single enzymes. Therefore, the optimal ratio of the complex enzyme is cellulase:pectinase:hemicellulase = 2:1:2. From the above experiments, it can be seen that the enzymatic effect of the combination of cellulase, pectinase, and hemicellulase is better than that of using one of them alone, and higher polysaccharide yields can be obtained.

### 2.2. Response Surface Assay

#### 2.2.1. Single−Factor Test

(1) The effect of enzyme dosage on polysaccharide yield

**Figure 2:**
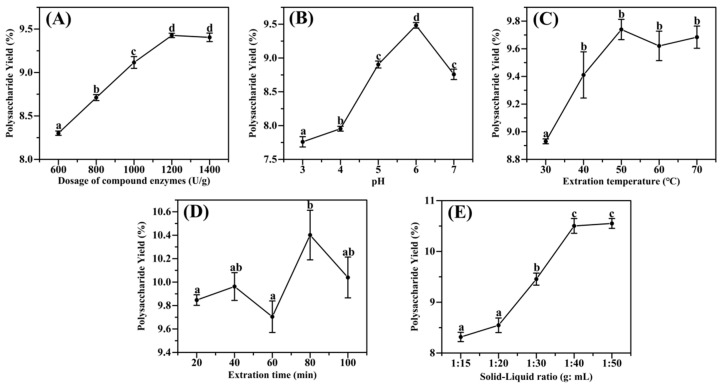
The effect of enzymatic conditions on the extraction rate of polysaccharides from *A. senticosus*: (**A**) the amount of enzymes; (**B**) the pH; (**C**) the extraction temperature; (**D**) the extraction time; (**E**) the solid–liquid ratio. Note: a, b, c and d: represent the significance of the difference within the graph. Containing the same letter means the difference is not significant (*p* > 0.05).

The amount of compound enzyme addition is an essential factor affecting the extraction rate, and the amount of compound enzyme addition affects the polysaccharide yield. Therefore, the addition amount was changed based on the compound enzyme ratio determined by the orthogonal test in order to examine the effect of compound enzyme addition on the yield of polysaccharides, as shown in Figure 2A. When the addition of the enzyme complex was increased from 600 U/g to 1200 U/g, the enzyme decreased the mass transfer resistance for polysaccharide solubilization in the plant cells by increasing the contact opportunity with the *Acanthopanax* powder and increased the polysaccharide yield. When it was increased to 1400 U, the polysaccharide yield slowed down. Within a certain range, the conversion rate of the substrate depends on the enzyme concentration; the greater the enzyme concentration, the chance of contact between the enzyme and the cell wall of the substrate plant increases, and the rate of polysaccharide yield increases accordingly. However, with excess enzyme, the excess enzyme reduces the chance of collision between the enzyme and the substrate, resulting in a lower reaction rate and a slower increase in the rate of polysaccharide yield. This finding matches up with other researchers’ reports on polysaccharide extraction [21,22]. From the viewpoint of the effectiveness and cost of enzymatic digestion, the optimal amount of enzyme addition for the complex enzyme is 1200 U/g.

(2) The effect of pH on polysaccharide yield

The pH can strongly affect the conformation and activity of the enzyme [23]. Therefore, it is vital to examine pH when investigating the optimal conditions for extracting *Acanthopanax* polysaccharides by complex enzymes. A slight increase in polysaccharide extraction was observed when the pH of the buffer was changed from 3 to 4; however, when it was changed from 4 to 6, the extraction rate increased considerably, and the highest polysaccharide yield of 9.48% was obtained at pH 6 (Figure 2B). This was followed by a significant decreasing trend, which may be due to a decrease in enzyme activity at higher pH [24]. It was shown that pH 6 was the optimal value for the extraction of *Acanthopanax* polysaccharides by the complex enzyme.

(3) The effect of enzymatic digestion temperature on polysaccharide yield

Since the solubility of polysaccharides increases at higher temperatures, the extraction rate may increase with increasing extraction temperature, and there is an optimal temperature for the enzyme to act [8]. Therefore, five temperature points between 30 °C and 70 °C were selected for the test. The results showed that the polysaccharide yield gradually increased when the extraction temperature was increased from 30 °C to 50 °C, indicating that the appropriate increase in temperature could enhance the extraction rate of polysaccharides. When the temperature exceeded 50 °C, it did not reflect higher polysaccharide yields but decreased, possibly due to partial inactivation or denaturation of the complex enzyme at too high a temperature [25]. This study’s best enzyme treatment temperature was 50 °C.

(4) The effect of extraction time on polysaccharide yield

The processing time is also essential for extracting polysaccharides from plant material. It will impact the final concentration of polysaccharides in the extract, extraction efficiency, and energy cost [26]. In this investigation, extraction time was from 20 min to 80 min, and the polysaccharides yield increased to 10.40% at 80 min (Figure 2D). Subsequently, the time was extended to 100 min, and the polysaccharide yields did not change much and decreased slightly. It may be because the substrate has been entirely enzymatically cleaved by the complex enzyme within the time of 80 min, and all the polysaccharides were released, and further extension of the time may cause some of the polysaccharides to be hydrolyzed [27]. For all subsequent studies, 80 min was the best enzyme extraction time.

(5) The effect of solid–liquid ratio on polysaccharide yield

The degree of enzyme action is influenced by the ambient temperature and pH and is related to the contact area between the enzyme and the substrate. Consequently, an adequate volume increases the contact area between the enzyme and substrate, thereby facilitating the enzyme’s function. According to Figure 2E, when the volume was expanded from 15 mL to 40 mL, the extraction rate kept increasing, probably because increasing the ratio of water to raw material increases the diffusivity of the solvent into the cells and enhances the desorption of polysaccharides from the particles [28]. The maximum 10.50% was reached at an extraction volume of 40 mL. Continue to increase the volume to 50 mL, and the extraction rate no longer increases, probably because too much solvent can cause excessive raw material swelling to adsorb polysaccharides [29]. Therefore, 40 mL was selected as the optimal extraction volume for this experiment.

#### 2.2.2. Model Fitting Analysis

Based on a single−factor test and Box–Behnken design principles, a four−factor, three−level response surface test was developed to optimize the ASPS extraction conditions. Solvent pH (A), enzyme treatment temperature (B, °C), enzyme treatment time (C, min), and solid–liquid ratio (D, g/mL) were used as independent variables, and the extraction rates of ASPSs (Y, %) were used as a 29−group response variable. The results are listed in Table 2. The following regression model was derived by fitting a quadratic regression to the data in Table 3 using Design Expert 13 software.

Y = 10.03 − 0.17A − 0.1983B − 0.1025C + 0.8358D − 0.385AB − 0.015AC − 0.305AD + 0.0475BC − 0.6075BD − 0.325CD − 0.7478A^2^ − 0.1528B^2^ − 0.1366C^2^ − 0.4391D^2^ (1)

where Y is the ASPS yield, A is the solvent pH, B is the enzyme treatment temperature, C is the enzyme treatment time, and D is the solid–liquid ratio; A^2^, B^2^, C^2^, and D^2^ are the quadratic coefficients of A, B, C, and D; and AB, AC, AD, BC, BD, and CD are the interaction coefficients between A, B, C, and D.

One−way variance was performed on the data collated in Table 4 (shown in Table 5). The model F-value is 30.7, which is highly significant (*p* < 0.01), and the *p*-value of the out−of−fit term is 0.3261, which is not significant (*p* > 0.05), showing that the equation is similar to the actual data. The above results support the model’s utility, which can be selected for the optimal experimental design of polysaccharide extraction conditions. The R^2^ result for the number of correlation coefficients was 0.8203, showing that the model adequately described the relationship between the four parameters and the polysaccharide extraction rate. The adjusted R^2^ (Radj^2^ = 0.9369) result suggests that the created model is credible and that unpredictable factors account for only 0.1% of the response variable. According to the significance criteria, it can be concluded that the primary term D and the interaction term BD reached a highly significant (*p* < 0.001) level, and the secondary terms A² and D² reached an important (*p* < 0.001) level. Based on the F-value, it can be seen that the level of impact of each factor on the yield of polysaccharides is solid–liquid ratio > extraction temperature > pH > extraction time.

#### 2.2.3. Analysis of Response Surface Plots

**Figure 3:**
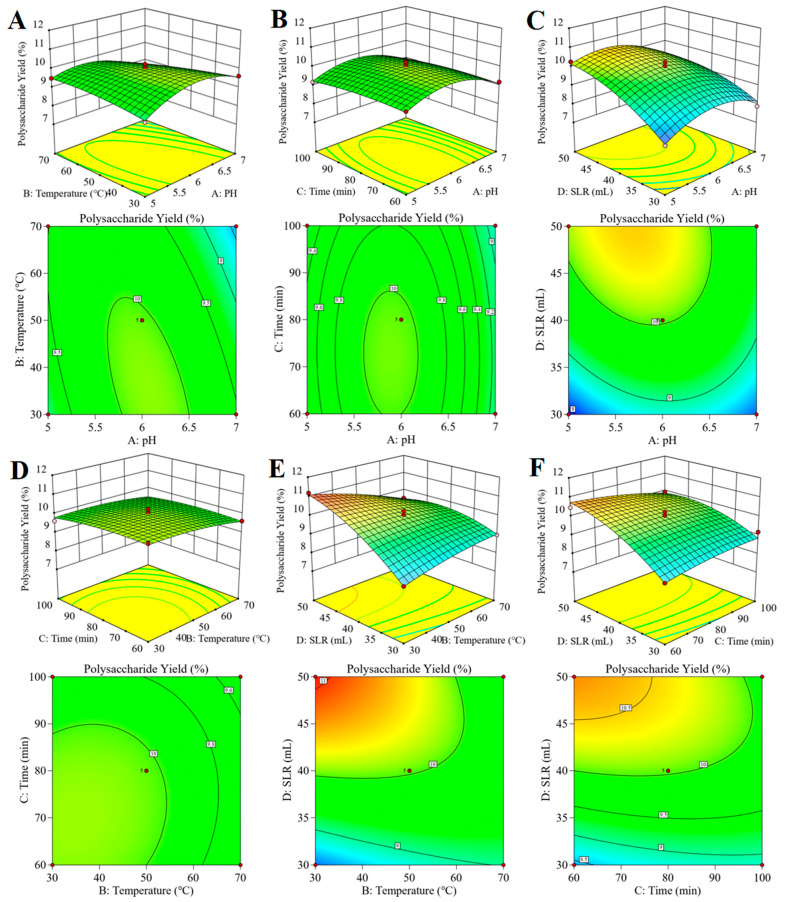
The 3D response surface plots and contour plots (**A**–**F**) of the influence of interactions of various factors on the extraction rate of ASPS. Note: SLR is the solid–liquid ratio.

The response surface three−dimensional curve, as shown in Figure 3, can visually show the trend of the influence of each factor on the response value. The steeper the curve trend in the response surface plot, the more significant the impact of the factor on the polysaccharide yield and the smaller the influence if the movement is smooth. The shape of the contour line reflects the degree of interaction between the two factors; an ellipse indicates a significant interaction between the two factors, while the opposite is true if it is circular. As can be seen from Figure 3A,C, the yield of ASPS varies significantly with the pH–temperature and pH–solid–liquid ratio, with larger curves and elliptical contours suggesting that the interaction of these two factors has a significant impact on the yield of ASPS. The upper panel of Figure 3B,D shows that the contours are flatter, tending to be planar, indicating that the pH–time and temperature–time interactions have almost no effect on the ASPS yields. The values on the response surface contours indicate the extraction rate in the horizontal and vertical coordinate condi-tions. From Figure 3E,F, it can be seen that the color of the response surface pictures changed from blue to red from right to left, indicating that the mass of the extract was gradually increasing. The response surface’s graph was steeper, showing that the temperature–liquid and time–liquid ratios had significant effects on the ASPS yield. These findings match Table 5’s variance analysis.

#### 2.2.4. Model Validation

The extraction conditions were further optimized based on model equation fitting predictions combined with the results of the one−way and orthogonal assays described above (Cellulase:Pectinase:Hemicellulase = 2:1:2, Complex: 1200 U/g). The optimized polysaccharide extraction conditions were pH 6.939, extraction temperature 30 °C, extraction time 60 min, and material−to−liquid ratio 1:50 (g:mL), and the predicted extraction rate under these conditions was 10.692%. Considering the practical operation, the pH was adjusted to 7 in the experiment, other states were kept unchanged, and three parallel extraction tests were performed to obtain an average polysaccharide extraction rate of 10.53 ± 0.682%. The relative error between the model and the predicted value is small, which shows that the model fits well and verifies the reliability of the model.

### 2.3. Ethanol−Graded Precipitation of Polysaccharides

The polysaccharide yields, total sugars, reducing sugars, proteins, and glucuronic acid contents of each fraction (ASPS−40, ASPS−60, and ASPS−80) were determined according to the above measurement methods. The molecular weights and monosaccharide compositions of the three fractions were measured and analyzed, and the results are shown in Table 4.

According to the results in Table 4, the yields of ASPS−40, ASPS−60, and ASPS−80 are 29.17 ± 0.57%, 45.60 ± 0.87%, and 22.92 ± 0.33%, respectively, after ethanol graded−precipitation. ASPS−40 contains 41.81 ± 2.46% total sugars, 5.51 ± 2.09% reducing sugars, and 2.56 ± 0.04% galacturonic acid, but no proteins; 40.13 ± 0.88% total sugars, 34.49 ± 0.44% reducing sugars and 4.71 ± 0.16% proteins in ASPS−60, and ASPS−60 do not contain galacturonic acid; The ASPS−80 contains 67.18 ± 0.31% total sugars, 25.17 ± 0.69% reducing sugars and 4.22 ± 0.17% protein, and ASPS−80 do not contain galacturonic acid. It can be seen that most of the reducing sugars with relatively small molecular weights are found in ASPS−60 and ASPS−80. ASPS−40 consists mainly of arabinose, glucose, galactose, and fructose with molar ratios of 0.034, 0.702, 0.023, 0.242, and the average molecular weight is determined to be 11.762 kDa; ASPS−60 consists mainly of glucose and fructose with molar ratios of 0.716, 0.284 and the average molecular weight is determined to be 7.890 kDa; ASPS−80 consists mainly of glucose and fructose with a molar ratio of 0.656, 0.344, and the molecular weight is determined to be 6.433 kDa. Extraction by complex enzymes and graded precipitation treatment by ethanol can cause degradation, grafting, and cross−linking reactions of polysaccharides, changing their size and spatial conformation and affecting their biological activity [30,31].

### 2.4. Fourier Transform Infrared (FT−IR) Analysis

**Figure 4:**
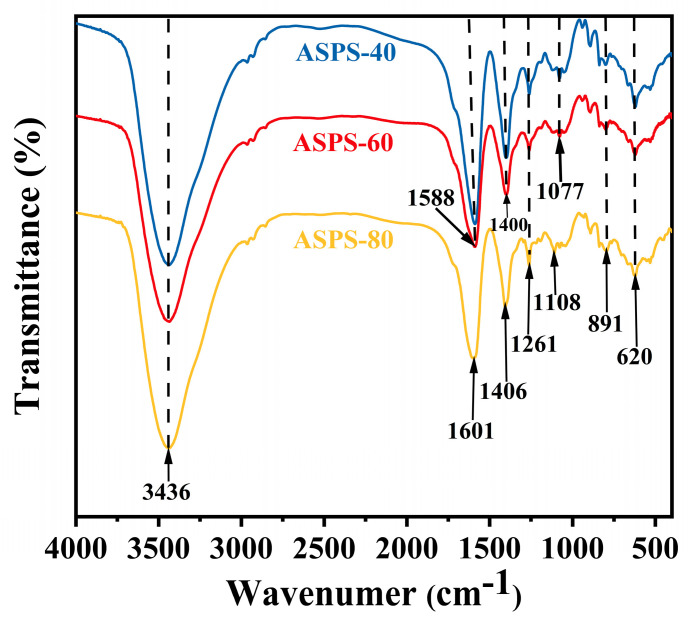
FT−IR images of ASPS−4, ASPS−60, ASPS−80.

The infrared spectra (Figure 4) show the main functional groups of the three polysaccharides. The peak appearing at 3436 cm^−1^ is indexed to the −OH stretching vibration [16], and relatively weak absorption peaks at 1588 cm^−1^ and 1601 cm^−1^ indicate the presence of COO−, indicating a small amount of glyoxylate in the polysaccharide [32]. The small absorption peaks appearing at 1400 cm^−1^ and 1406 cm^−1^ are variable angle vibrational peaks of C−H, the absorption peak at 1261 cm^−1^ represents a double bond = SO symmetric stretching vibrational peak [33], the absorption peaks at 1077 cm^−1^ and 1108 cm^−1^ suggest the presence of pyranose rings [34], the fact of absorption peaks at 891 cm^−1^ indicates the presence of β−glycosidic bonds [35], and the vibrational peaks at and after 620 cm^−1^ are fingerprint regions. These absorption peaks are typical for polysaccharides, indicating that the primary structure of polysaccharides extracted by complex enzymes and obtained by ethanol precipitation is not damaged.

### 2.5. UV Assay

**Figure 5:**
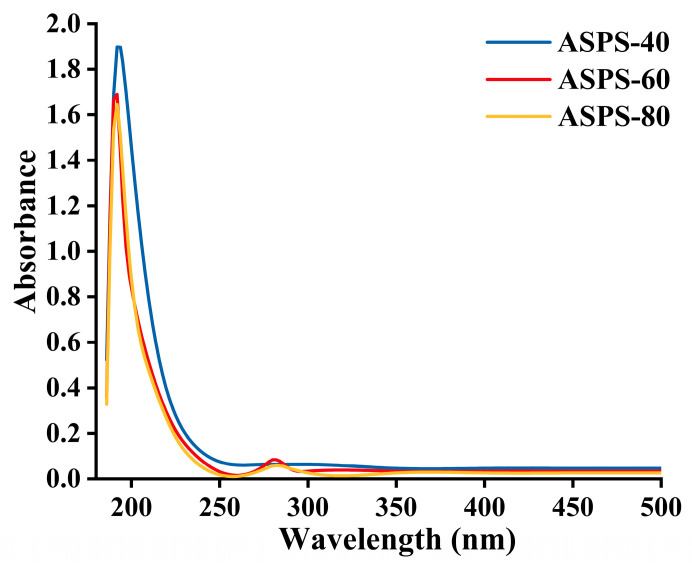
UV spectra of ASPS−40, ASPS−60, ASPS−80.

As shown in Figure 5, the scanned UV spectra of the three polysaccharides showed that two polysaccharides, ASPS−60 and ASPS−80, showed weak absorption peaks at 280 nm, indicating that these two polysaccharide fractions contained a small amount of protein, which was consistent with the protein results measured in Table 6.

### 2.6. TGA Analysis

**Figure 6:**
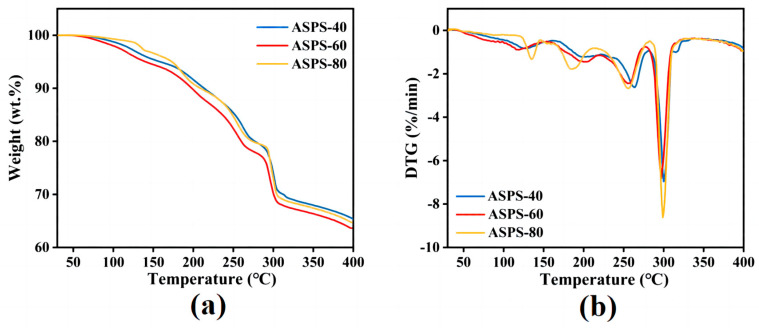
TGA (**a**) and DTG (**b**) analysis images of ASPS−40, ASPS−60, and ASPS−80.

TGA was used to characterize the thermal properties of the polysaccharides. As shown in Figure 6, the weight change trends of the three polysaccharides are in the range of 30–400 °C. The weight loss of polysaccharides has three different stages, and the first stage of weight loss occurs at 75–150 °C, with 5.12%, 5.23%, and 3.72% loss in ASPS−40, ASPS−60, and ASPS−80, respectively, mainly due to the evaporation of free and bound water. The second stage occurs at 150–280 °C and is the leading weight loss stage, with ASPS−40, ASPS−60, and ASPS−80 losing 11.36%, 16.87%, and 16.48% of the total weight, respectively. In the last step, it can be seen from the DTG curve that 300 °C is the fastest temperature for weight loss rate, and the weight loss rate slows down significantly when the temperature is more significant than 300 °C. The residual masses of ASPS−40, ASPS−60, and ASPS−80 after loss are 65.36%, 63.64%, and 64.74%, respectively. It shows that the thermal stability of the ASPS is good.

### 2.7. SEM Assay

**Figure 7:**
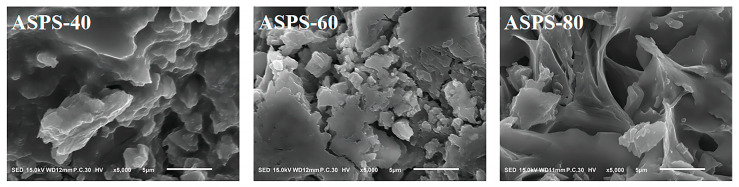
SEM images of ASPS−40, ASPS−60, and ASPS−80 (5000×).

SEM is an effective method for measuring the surface shape of polymers, including polysaccharides. The 5000× electron microscopy images of ASPS−40, ASPS−60, and ASPS−80 are presented in Figure 7. It can be observed that the surface of ASPS−40 is rough and has irregular bumps; ASPS−60 is a lumpy structure with different sizes and sharp edges; ASPS−80 has a smooth texture and a “drawn” shape. The results showed that the three polysaccharides with different molecular weights obtained by ethanol−graded precipitation have other morphological structures.

### 2.8. X-ray Diffraction (XRD) Analysis

**Figure 8:**
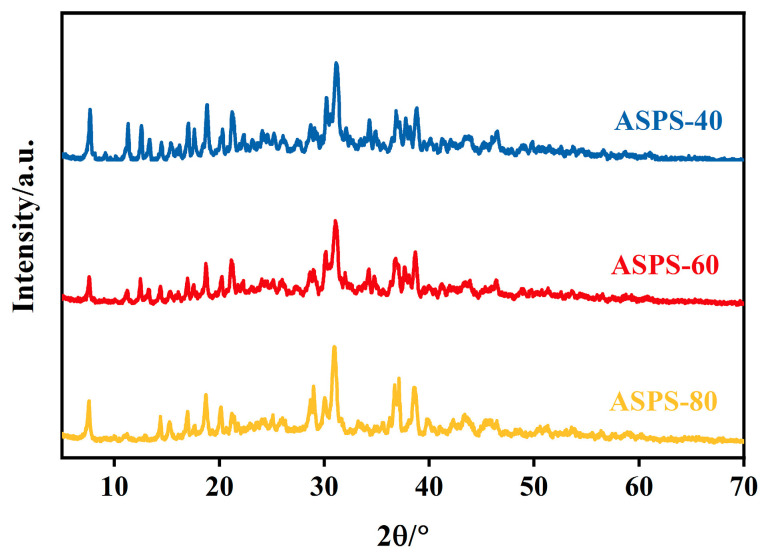
XRD spectra of ASPSs.

X-ray diffraction (XRD) is a significant technique utilized for the characterization of polymer crystal structures, enabling deeper analysis of polysaccharide structures [36]. In general, crystalline materials appear as sharp narrow diffraction peaks, whereas amorphous components appear as broad peaks [37]. The X-ray diffraction (XRD) results of the ASPSs are shown in Figure 8. This indicates that the overall crystallinity of ASPSs is low and there are no obvious peaks. These results suggest that the internal structure of ASPSs is amorphous and that ethanol−graded precipitation affected the structure of ASPSs.

### 2.9. Congo Red and I_2_−KI Test

**Figure 9:**
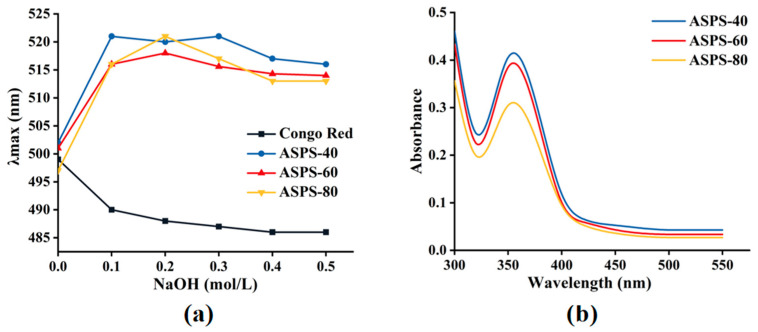
Structural characterization of ASPS−40, ASPS−60, and ASPS−80. (**a**) The λmax of Congo red + polysaccharides at various NaOH concentrations; (**b**) UV−vis spectrum in the presence of I_2_−KI.

Congo red is a quick approach for identifying polysaccharide conformations, which changes its maximum apparent absorption peak in the presence of helical structures in polysaccharides [38]. Figure 9a shows the results of the Congo red test for the three *Acanthopanax* polysaccharide fractions, and the maximum UV absorption peaks of ASPS−40, ASPS−60, and ASPS−80 showed a red shift compared to the Congo red control, indicating the presence of triple helix conformation for all three polysaccharide fractions [39], which is consistent with the findings of Mohammed et al. [40]. The different trends in the maximum absorption peaks of the three polysaccharide fractions in NaOH solutions of different concentrations showed that the stability of the conformation is related to the size of the molecular weight [41], and the maximum absorption peaks of the polysaccharides gradually decreased when the NaOH concentration gradually increased, indicating that the hydrogen bonds were broken by the alkaline solution [42].

The I_2_−KI test can be used to determine whether polysaccharides include relatively lengthy, multi−branched structures. I_2_′s absorption summit changes from 350 nm to 565 nm when polysaccharides with fewer branches and shorter side chains bind to it [43]. As shown in Figure 9b, the absence of absorption peaks near 565 nm in ASPS−40, ASPS−60, and ASPS−80 solutions in the figure indicates that all three fractions of polysaccharides have a complex structure with more branches and longer side chains in the backbone and no starch [44].

### 2.10. In Vitro Antioxidant Activity Assay

#### 2.10.1. ABTS Free Radical Scavenging Capacity

**Figure 10:**
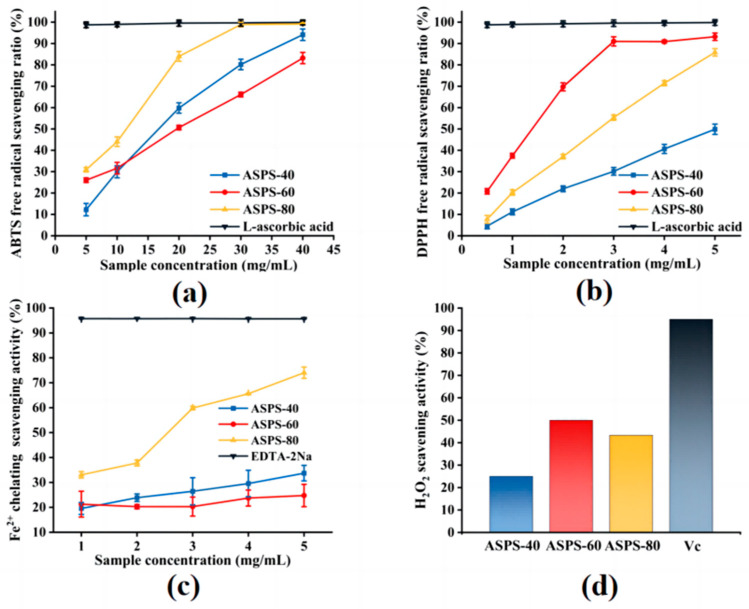
Antioxidant test of ASPS−40, ASPS−60, and ASPS−80. (**a**) ABTS radical scavenging activities; (**b**) DPPH radical scavenging activities; (**c**) H_2_O_2_ radical scavenging activities; (**d**) Fe^2+^ chelating activities.

In the ABTS radical scavenging assay (Figure 10a), the IC_50_ of ASPS−40, ASPS−60, and ASPS−80 was 18.11 mg/mL, 19.86 mg/mL, and 10.53 mg/mL, respectively, and the scavenging ability of the three polysaccharide fractions was: ASPS−80 > ASPS−40 > ASPS−60. The scavenging of ABTS radicals by all three polysaccharide fractions increased rapidly with increasing concentration, with ASPS−80 showing a more vital scavenging ability, indicating that the smaller the molecular weight of the polysaccharide, the stronger the scavenging capacity of ABTS radicals, which is consistent with previous findings [45]. At 40 mg/mL polysaccharide concentration, the scavenging rates of ABTS radicals by ASPS−40, ASPS−60, and ASPS−80 were 94.14%, 83.19%, and 99.10%, respectively.

#### 2.10.2. DPPH Free Radical Scavenging Capacity

In the DPPH radical scavenging assay (Figure 10b), the IC_50_ of ASPS−40, ASPS−60, and ASPS−80 were 5.174 mg/mL, 1.172 mg/mL, and 2.355 mg/mL, respectively, and the scavenging ability of the three polysaccharide fractions was: ASPS−60 > ASPS−80 > ASPS−40. The scavenging rate of DPPH radicals by ASPS−40 and ASPS−80 showed a linear increase with increasing concentration, and the increased slope is more significant for ASPS−80. Conversely, the scavenging rate of DPPH radicals by ASPS−60 increased rapidly with increasing concentration and then leveled off. The IC_50_ of ASPS−60 is the smallest, indicating that its ability to scavenge DPPH radicals was the strongest, probably related to its higher reducing sugar content. At the 5 mg/mL polysaccharide concentration, the maximum DPPH radical scavenging rates of ASPS−40, ASPS−60, and ASPS−80 are 51.52%, 91.89%, and 86.73%, respectively.

#### 2.10.3. Fe^2+^ Chelating Activity of ASPSs

Transition metals were stronger prooxidants due to their higher reactivity and could aggravate cellular damage. Fe^2+^ chelating is an antioxidant mechanism and commonly used in antioxidant research [46]. As shown in Figure 10c, the Fe^2+^ chelating activity of ASPS−40, ASPS−60, and ASPS−80 increased from 19.55%, 21.26%, and 33.05% to 33.72%, 24.75%, and 74.03%, respectively, when the concentration of three polysaccharides increased from 1 to 5 mg/mL. The IC_50_ values of ASPS−40, ASPS−60, and ASPS−80 are 9.033 mg/mL, 25.006 mg/mL, and 2.618 mg/mL. Interestingly, ASPS−80 shows a strong chelating ability for Fe^2+^, while ASPS−40 and ASPS−60 are weaker, which might be related to the smaller molecular weight of ASPS−80 [47].

#### 2.10.4. H_2_O_2_ Radical Scavenging Activity Assay

In the H_2_O_2_ radical scavenging assay (Figure 10d), at a concentration of 5 mg/mL, with the same concentration of Vc as a control, it can be seen that the scavenging ability of ASPS−40, ASPS−60, and ASPS−80 for H_2_O_2_ is 25.00%, 50.00%, and 43.33%, respectively, whereas the scavenging rate of Vc for H_2_O_2_ reached 95.00%. This indicated that all ASPSs have a weak scavenging ability for H_2_O_2_, and the test is stable and repeated three times with almost no error.

## 3. Materials and Methods

### 3.1. Materials and Chemicals

*A. senticosus* was obtained from Limin Pharmacy (Liaocheng, China), ground into a powder using a grinder (H8422, Hebei Huicai Technology Co., Ltd., Hebei, China), and sieved through a 60−mesh sieve. Cellulase (1 × 10^4^ U/g), Pectinase (3 × 10^4^ U/g), and Hemicellulase (2 × 10^5^ U/g) were purchased by Shanghai Macklin Biochemical Technology Co., Ltd. (Shanghai, China). All other reagents were at least of analytical grade.

### 3.2. Preparation of A. senticosus Polysaccharide

The powder (1.0 g) was precisely weighed and placed in a clay−shaped bottle. The enzyme quantity, solid–liquid ratio, enzymatic digestion time, enzymatic digestion temperature, and pH were analyzed in a constant−temperature water bath boiler. After enzymatic digestion, the solution was heated to 95 °C for 5 min to activate the enzyme. Filtration was performed with filter paper, and the filtrate was collected to obtain the polysaccharide extracts. The extraction solution was added 95% ethanol to reach an 80% ethanol final concentration and then left at 4 °C overnight. After the alcoholic precipitation, the polysaccharide extract from *A. senticosus* was obtained by centrifugation and drying. The polysaccharide yields were determined using the following equation:

(2) $$\mathrm{Polysaccharide}\;\mathrm{yield}\%\;\left(W/W\right)=\frac{\mathrm{weight}\;\mathrm{of}\;\mathrm{polysaccharides}\;}{\mathrm{weight}\;\mathrm{of}\;\mathrm{AS}\;\mathrm{root}\;\mathrm{powder}}\times 100$$

### 3.3. Experimental Design

#### 3.3.1. Single Factor Experiment

In order to determine the optimal dosage of each enzyme, tests were carried out according to the optimal enzymatic conditions for the three enzymes (pectinase temperature 50 °C, pH 3.4, cellulase temperature 50 °C, pH 5, hemicellulase temperature 30 °C, pH 5). A total of 1.0 g of *Acanthopanax* powder was precisely weighed to screen the amount of cellulase addition (25 U/g, 50 U/g, 100 U/g, 200 U/g, 400 U/g), pectinase addition (12.5 U/g, 25 U/g, 50 U/g, 100 U/g), hemicellulase addition (25 U/g, 50 U/g, 100 U/g, 200 U/g, 400 U /g), respectively.

#### 3.3.2. Compound Enzyme Ratios Optimized by Orthogonal Experiment

Based on the above single−factor test results, we used the orthogonal test L_9_(3^3^) to design the optimal concentration ratios of cellulase, pectinase, and hemicellulase. The fixed extraction conditions were as follows: the solid–liquid ratio was 1:40 (g/mL), the extraction temperature was 50 °C, the extraction time was 60 min, and the pH was 5.0. The experimental design is shown in Table 5. The experiment was conducted in nine groups of parallel tests, and the extracts were collected and treated according to the method in 3.2.

#### 3.3.3. Single−Factor Experiment

The 1.0 g of *Acanthopanax* powder was weighed precisely. The five factors were investigated according to the optimal compound enzyme ratio determined by the orthogonal test, which included the amount of compound enzyme (600 U/g, 800 U/g, 1000 U/g, 1200 U/g, 1400 U/g), enzymatic pH (3, 4, 5, 6, 7), enzymatic temperature (30 °C, 40 °C, 50 °C, 60 °C, 70 °C), enzymatic time (20, 40, 60, 80, 100 min), solid–liquid ratio (1:15, 1:20, 1:30, 1:40, 1:50). The polysaccharide yield was determined according to the steps in 3.2, and the extraction was conducted in triplicate.

#### 3.3.4. Response Surface Test

Based on single−factor tests, the original range of independent variables was chosen. A four−factor, three−level response surface experiment was designed utilizing the response surface methodology (RSM) and Box–Behnken design (BBD). The enzyme treatment pH, temperature, time, and solid–liquid ratio were used as independent variables, and the extraction rate of polysaccharides was used as the dependent variable. These are shown in Table 6.

#### 3.3.5. Validation Tests

Three repetitions of enzyme−assisted aqueous extraction of Acanthopanax polysaccharides using the optimal conditions derived from the response surface.

### 3.4. Ethanol−Graded Precipitation of Polysaccharides

The optimum technique for extracting *Acanthopanax* polysaccharides was utilized for the extraction procedure based on the experiments mentioned above. The extraction liquid was mixed with 95% ethanol until it had a final concentration of 80% ethanol in water, and then put in a refrigerator at 4 °C for 12 h. Remove the supernatant to obtain crude polysaccharide. By adding the right amount of distilled water, the polysaccharide was dissolved. It was then deproteinized with the Sevage method, depigmented with H_2_O_2_, and then precipitated with 80% ethanol to get pure polysaccharide. Add distilled water to dissolve the purified polysaccharide, add 95% ethanol to the ethanol final concentration of 40%, rest for 12 h at 4 °C in the refrigerator, centrifuge at 3000 r/min for 5 min, and the supernatant was separated, the precipitate was placed at 105 °C and dry for 1.5 h to obtain ASPS−40. The supernatant was added with 95% ethanol to 60% ethanol concentration and left to stand for 12 h at 4 °C in the refrigerator. The subsequent operation was the same as above to obtain ASPS−60 and ASPS−80.

### 3.5. Component Analysis

The phenol−sulfuric acid method was employed to measure the total amount of sugar, with glucose serving as a reference [48]. The Coomassie Brilliant Blue G−250 staining method was used to determine the protein content with bovine serum albumin as standard [49]. The glucuronide content was determined by the m−hydroxyphenyl colorimetric method using D−glucuronide as a standard [50].

### 3.6. Molecular Weight Determination

HPGPC determined the molecular weights of ASPS−40, ASPS−60, and ASPS−80. The samples and standards were carefully weighed, the samples were made into a 5 mg/mL solution, spun at 10,142× *g* for 10 min, and the supernatant was filtered through a 0.22 μm microporous filter membrane. The sample was then put into an injection tube with 1.8 mL. The chromatographic column used in this study was a BRT105−104−102 tandem gel column with dimensions of 8 × 300 mm. The mobile phase employed was a 0.05 M NaCl solution, and the flow rate was set at 0.6 mL/min. The column temperature was maintained at 40 °C throughout the experiment. A volume of 25 μL was injected into the column, and the detector used was the Differential detector RI−10A.

### 3.7. Monosaccharide Composition Analysis

The 5 mg of sample was placed in an ampoule, 3 M trifluoroacetic acid (TFA) 2 mL added, and hydrolyzed at 80 °C for 3 h. The acid hydrolysis solution was accurately aspirated, transferred to a tube with nitrogen blowing, and dried. 5 mL of water, vortexed to mix and centrifuged at 10,142× *g* for 5 min. The supernatant was taken into the ion chromatograph for analysis.

The chromatographic column used in this study was the Dionex Carbopac TM PA20, with dimensions of 3 × 150 mm. The mobile phase consisted of three components: A, which was composed of H_2_O; B, which contained 15 mM NaOH; and C, which consisted of a mixture of 15 mM NaOH and 100 mM NaOAc. The flow rate of the mobile phase was set at 0.3 mL/min. A volume of 25 µL was injected into the column. The column temperature was maintained at 30 °C throughout the experiment. An electrochemical detector was employed to detect and analyze the compounds.

### 3.8. Fourier Transform Infrared (FT−IR) Analysis

The dried sample (1.0 mg) was mixed with 150 mg of KBr powder, uniformly ground, and pressed, and then FT−IR spectra were recorded from 400 to 4000 cm^−1^ using a Nicolet iS10 FT−IR spectrometer (Thermo Fisher Shanghai, China).

### 3.9. UV−Vis Spectroscopy

The UV−vis spectra of the polysaccharide solutions were recorded in the range of 200–500 nm using a UV−vis spectrophotometer (Nano Ready F−1100 Shanghai Metash Instruments Co., Ltd., Shanghai, China).

### 3.10. Thermogravimetric Analysis

Thermogravimetric analysis was performed in the temperature range of 30 °C–400 °C with a scanning speed of 10 °C/min using a NETZSCH 209 F1 instrument German, N_2_ environment.

### 3.11. SEM Analysis

This study compared the morphological characteristics of three different molecular weights polysaccharide by uniformly attaching the dried powder to the sample stage and spraying gold. Imaging was performed using a scanning electron microscope (Tokyo, Japan, Electron Optics Laboratory jsm−it100) at an accelerating voltage of 15.0 kV and a magnification of 5000×.

### 3.12. X-ray Diffraction (XRD) Analysis of ASPSs

The X-ray diffractograms were taken with a D8 Advance X-ray diffractometer utilizing copper lamp radiation (Cu−Ka) at 15 mA and 40 kV, 2°/min, 5–70 °C.

### 3.13. Congo Red and I_2_−KI Test

Congo red (80 μmol/L, 2.0 mL) was mixed with polysaccharide solution (2.0 mg/mL, 2.0 mL), and a specific volume of NaOH solution (1 mol/L) was added to make the final concentration of NaOH between 0.0 mol/L and 0.5 mol/L. After the reaction at room temperature for 5 min, the maximum absorption wavelength (λmax) was measured using a UV−Vis spectrophotometer (Nano Ready F−1100 Shanghai Metash Instruments Co., Ltd., Shanghai, China).

The I_2_−KI test involved mixing I_2_−KI (0.2%, 8.0 mL) with polysaccharide (2.0 mg/mL, 2.0 mL) and recording the absorbance at 300–700 nm with a UV−Vis spectrophotometer (Nano Ready F−1100 Shanghai Metash Instruments Co., Ltd., Shanghai, China).

### 3.14. Analysis of Antioxidant Activity

#### 3.14.1. ABTS Radical Scavenging Activity Assay

A described approach was slightly modified to test the abilities of polysaccharides to scavenge ABTS radicals [51]. The samples (4 mL) at different concentrations (5, 10, 20, 30, 40 mg/mL) were mixed with 5.8 mL of ABTS working solution. Then, the mixtures were shaken and kept at room temperature for 30 min in a dark room. The absorbance of the mixture was measured at 734 nm with a UV spectrophotometer. The same concentration of Ascorbic acid (Vc) was used as the positive control. Anhydrous ethanol was used as a blank control. The ABTS scavenging activity was calculated using the following formula:

(3) $$\mathrm{Scavenging}\;\mathrm{activity}\;\left(\%\right)=\frac{A_{1}-\left(A_{2}-A_{3}\right)}{A_{1}}\times 100$$

where A_1_, A_2_, and A_3_ were the absorbances of the ABTS blank, sample, and control, respectively.

#### 3.14.2. DPPH Radical Scavenging Activity Assay

Polysaccharides were tested for their DPPH radical−scavenging activities using a previously reported slightly modified [52]. In brief, 2.0 mL of samples at different concentrations (0.5, 1, 2, 3, 4, 5 mg/mL) was added to 2.0 mL of a methanolic solution (0.2 mM) of DPPH. The mixture was shaken and mixed and then reacted in the dark for 30 min. The absorbance of the mixture was measured at 517 nm. The same concentration of Ascorbic acid (Vc) was used as the positive control. Anhydrous ethanol was used as a blank control. The experiment was conducted in triplicate. This scavenging activity was calculated by following the equation.

(4) $$\mathrm{Scavenging}\;\mathrm{activity}\;\left(\%\right)=\frac{B_{1}-\left(B_{2}-B_{3}\right)}{B_{1}}\times 100$$

where B_1_, B_2_, and B_3_ were the absorbances of the DPPH control, sample, and blank, respectively.

#### 3.14.3. Fe^2+^ Chelating Activity

The Fe^2+^ chelating capabilities of ASPSs were mildly modified [53]. For each sample (400 μL), 50 μL of FeCl_2_ solution (2 mM), 100 μL of ferrozine solution (5 mM), and 2 mL of methanol were combined. The mixture was agitated well, reacted for 10 min at room temperature, and measured at 562 nm with ethylenediaminetetraacetic acid disodium salt as a positive control. The following formula calculated Fe^2+^ chelating activity.

(5) $${\mathrm{Fe}}^{2+}\;\mathrm{chelating}\;\mathrm{activity}\;\left(\%\right)=\frac{C_{1}-\left(C_{2}-C_{3}\right)}{C_{1}}\times 100$$

where C_1_, C_2_, and C_3_ were the absorbances of the control, sample, and blank, respectively.

#### 3.14.4. H_2_O_2_ Radical Scavenging Activity Assay

The ability to remove H_2_O_2_ was determined according to the reported method with minor modifications [54]. A solution was created with 1 mL of freshly made H_2_O_2_ (0.1 mmol L^−1^), 1 mL of diluted polysaccharide, 0.1 mL of ammonium molybdate (3% *w*/*v*), 10 mL of H_2_SO_4_ (2 mol L^−1^), and 7 mL of KI (1.8 mol L^−1^). The mixture was titrated with Na_2_S_2_O_3_ (5 mmol L^−1^) until the yellow color faded. For comparison, the removal capacity of L−ascorbic acid at the same sample concentration was determined. The removal activity was calculated according to the following equation:

(6) $$\mathrm{Scavenging}\;\mathrm{activity}\;\left(\%\right)=\frac{V_{0}-V_{1}}{V_{0}}\times 100$$

where, V_0_ was the volume of the Na_2_S_2_O_3_ solution used to titrate the control mixture and V_1_ was the titration volume of the sample−containing mixtures.

### 3.15. Statistical Analysis

The Box–Behnken experimental design was designed with Design−Expert 13, the graph was plotted with Origin 2021, and the data were analyzed with SPSS statistical software version 24.0. The results were shown as the mean ± standard deviation, and the data were analyzed using a one−way ANOVA.

## 4. Conclusions

The polysaccharides from *A. senticosus* had been extracted from the *Acanthopanax* root by enzymolysis−assisted hot water. The study showed that the content and extraction rate of *Acanthopanax* polysaccharides could be significantly improved. After optimization, the polysaccharide extraction rate from *A. senticosus* was 10.53 ± 0.682%, which was about 2.5 times higher than that of hot water extraction, and the polysaccharide content was directly affected by enzymolysis. Three polysaccharide fractions, namely, ASPS−40, ASPS−60, and ASPS−80, were obtained by ethanol−graded precipitation, and the three polysaccharide fractions differed significantly in total sugar, protein, and glyoxylate contents. ASPS−60 had the highest polysaccharide yield, ASPS−40 contained glyoxylate, and glucose and xylose were the main monosaccharides constituting the three polysaccharides. The FT−IR and TGA results showed that the three polysaccharides did not differ in structure and thermal stability, but SEM showed that the three polysaccharides had distinctly different microstructures. The XRD suggests that the internal structure of ASPSs is amorphous. The Congo red test proved that all three polysaccharides have a triple helix structure, and the I_2_−KI test confirmed that all three polysaccharides have longer branched chains and more side chains. The results of antioxidant tests conducted by four methods showed that the antioxidant activity of polysaccharides is not only related to the molecular weight size but also can be related to its composition and structure. This study can improve the yield of *Acanthopanax* polysaccharides and provide a research direction and reference for further development and utilization of *A. senticosus*.

## Figures and Tables

**Table 1:** Orthogonalization test of complex enzyme ratios.

No.	Cellulase (U/g)	Pectinase (U/g)	Hemicellulase (U/g)	Polysaccharide Yield (%)
1	800	100	200	8.43
2	100	100	400	7.91
3	400	200	400	9.07
4	800	200	100	8.57
5	400	50	200	8.43
6	400	100	100	8.81
7	100	200	200	8.40
8	100	50	100	8.14
9	800	50	400	9.04
K1	24.44	25.61	25.52	
K2	26.30	25.14	25.26	
K3	26.04	26.04	26.01	
k1	8.15	8.54	8.51	
k2	8.77	8.38	8.42	
k3	8.68	8.68	8.67	
R	0.62	0.30	0.25	

**Table 2:** Design and results for the response surface experiment.

Run	A	B	C	D	Polysaccharide Yield Y (%)	
					Real Value Y	Predicted Value Y’
1	−1	−1	0	0	9.03	9.12
2	0	0	−1	−1	8.41	8.40
3	0	1	1	0	9.27	9.49
4	0	−1	−1	0	10.16	10.09
5	−1	0	−1	0	9.46	9.41
6	0	0	−1	1	10.47	10.72
7	0	0	0	0	10.02	10.03
8	0	−1	0	−1	8.21	8.20
9	1	1	0	0	8.55	8.38
10	0	−1	0	1	11.19	11.08
11	1	0	0	1	9.11	9.21
12	−1	0	0	1	10.27	10.16
13	1	0	−1	0	9.20	9.10
14	0	0	0	0	10.05	10.03
15	0	1	−1	0	9.62	9.60
16	−1	1	0	0	9.50	9.49
17	0	1	0	−1	8.98	9.02
18	−1	0	1	0	9.20	9.23
19	0	0	1	−1	9.18	8.85
20	0	0	0	0	9.8	10.03
21	0	1	0	1	9.53	9.47
22	−1	0	0	−1	7.82	7.88
23	1	−1	0	0	9.62	9.55
24	0	0	0	0	10.25	10.03
25	0	0	0	0	10.05	10.03
26	1	0	1	0	8.88	8.86
27	1	0	0	−1	7.88	8.15
28	0	0	1	1	10.62	9.87
29	0	−1	1	0	9.62	9.79

A: pH; B: temperature; C: time; D: solid–liquid ratio.

**Table 3:** ANOVA for the response surface quadratic model.

Source	SS	DF	MS	F	Pr > F	Sig
Model	16.45	14	1.18	30.7	<0.0001	significant
A	0.3468	1	0.3468	9.06	0.0094	**
B	0.472	1	0.472	12.33	0.0035	**
C	0.1261	1	0.1261	3.29	0.091	
D	8.38	1	8.38	219.07	<0.0001	***
AB	0.5929	1	0.5929	15.49	0.0015	**
AC	0.0009	1	0.0009	0.0235	0.8803	
AD	0.3721	1	0.3721	9.72	0.0076	**
BC	0.009	1	0.009	0.2358	0.6347	
BD	1.48	1	1.48	38.58	<0.0001	***
CD	0.4225	1	0.4225	11.04	0.005	**
A^2^	3.63	1	3.63	94.79	<0.0001	***
B^2^	0.1515	1	0.1515	3.96	0.0665	
C^2^	0.121	1	0.121	3.16	0.0971	
D^2^	1.25	1	1.25	32.68	<0.0001	***
Residual	0.5358	14	0.0383			
Lack of Fit	0.4336	10	0.0434	1.7	0.3216	not significant
Pure Error	0.1021	4	0.0255			
Cor Total	16.99	28				

Note: SS: Sum of squares; DF: Degree of freedom; MS: Mean square; Pr: Probability; ** *p* < 0.01, *** *p* < 0.001; R^2^ = 0.8203, Adjusted R^2^ = 0.9369, Predicted R^2^ = 0.8436, C.V.% = 2.076.

**Table 4:** Physicochemical properties of ASPS−40, ASPS−60, and ASPS−80.

Physicochemical Properties	ASPS−40	ASPS−60	ASPS−80
Yields (w%) ^a^	29.167 ± 0.567	45.602 ± 0.866	22.917 ± 0.327
Total polysaccharides (w%) ^a^	41.811 ± 2.462	40.131 ± 0.881	67.182 ± 0.311
Reducing sugar (w%) ^a^	5.514 ± 2.089	34.491 ± 0.438	25.171 ± 0.686
Galacturonic acid (w%) ^a^	2.562 ± 0.041	− ^c^	− ^c^
Total proteins (w%) ^a^	− ^c^	4.708 ± 0.157	4.222 ± 0.165
Molecular weights (Mw, kDa)	11.762	7.890	6.433
Monosaccharide composition (mol%) ^b^			
Arabinose	0.034	− ^c^	− ^c^
Glucose	0.702	0.716	0.656
Galacturonic	0.023	− ^c^	− ^c^
Fructose	0.242	0.284	0.344

Data is represented by mean ± SD, which is the mean of three replicates. ^a^: Weight percentage. ^b^: Molar ratio. ^c^: Not detected.

**Table 5:** The orthogonal test designs.

No.	Cellulase (U/g)	Pectinase (U/g)	Hemicellulase (U/g)
1	800	100	200
2	100	100	400
3	400	200	400
4	800	200	100
5	400	50	200
6	400	100	100
7	100	200	200
8	100	50	100
9	800	50	400

**Table 6:** Box–Behnken experimental design.

Factors	Level		
	−1	0	1
A pH	5	6	7
B Temperature (°C)	30	50	70
C Time (min)	60	80	100
D Solid–liquid ratio (g/mL)	1:30	1:40	1:50

## Data Availability

Not applicable.

## Sample Availability

Available.

## References

[1] Yang S.B., Shan C.L., Ma X., Qin Y.J., Ju A.Q., Duan A.Y., Luan W.M., Zhang Y.N. (2021). Immunomodulatory effect of Acanthopanax senticosus polysaccharide on immunosuppressed chickens. Poult. Sci..

[2] Zhang Y., Zhang Y., Liu Z. (2021). Effects of Acanthopanax senticosus supplementation on innate immunity and changes of related immune factors in healthy mice. Innate Immun..

[3] Han J., Liu L., Yu N., Chen J., Liu B., Yang D., Shen G. (2016). Polysaccharides from Acanthopanax senticosus enhances intestinal integrity through inhibiting TLR4/NF−κB signaling pathways in lipopolysaccharide−challenged mice. Anim. Sci. J. Nihon Chikusan Gakkaiho.

[4] Meng Q., Pan J., Liu Y., Chen L., Ren Y. (2018). Anti−tumour effects of polysaccharide extracted from Acanthopanax senticosus and cell−mediated immunity. Exp. Ther. Med..

[5] Long L., Zhang H., Wang F., Yin Y., Yang L., Chen J. (2021). Research Note: Effects of polysaccharide−enriched Acanthopanax senticosus extract on growth performance, immune function, antioxidation, and ileal microbial populations in broiler chickens. Poult. Sci..

[6] Yang Y., Yang M., Zhou X., Chen H. (2022). Optimization of Extraction Process of Polysaccharides MAP−2 from Opuntia Milpa Alta by Response Surface Methodology and Evaluation of Its Potential as α−Glucosidase Inhibitor. Foods.

[7] Song Y., Sung S., Jang M., Lim T., Cho C., Han C., Hong H. (2018). Enzyme−assisted extraction, chemical characteristics, and immunostimulatory activity of polysaccharides from Korean ginseng (*Panax ginseng* Meyer). Int. J. Biol. Macromol..

[8] Nagendra Chari K., Manasa D., Srinivas P., Sowbhagya H. (2013). Enzyme−assisted extraction of bioactive compounds from ginger (*Zingiber officinale* Roscoe). Food Chem..

[9] Khamassi A., Dumon C. (2023). Enzyme synergy for plant cell wall polysaccharide degradation. Essays Biochem..

[10] Gao S., Yan S., Zhou Y., Feng Y., Xie X., Guo W., Shen Q., Chen C. (2022). Optimisation of enzyme−assisted extraction of Erythronium sibiricum bulb polysaccharide and its effects on immunomodulation. Glycoconj. J..

[11] Shi M., Shi Y., Jin H., Cao J. (2023). An efficient mixed enzymes−assisted mechanical bio−extraction of polysaccharides from Dendrobium officinale and determination of monosaccharides by HPLC−Q−TOF/MS. Int. J. Biol. Macromol..

[12] Wang L., Cheng L., Liu F., Li T., Yu Z., Xu Y., Yang Y. (2018). Optimization of Ultrasound−Assisted Extraction and Structural Characterization of the Polysaccharide from Pumpkin (*Cucurbita moschata*) Seeds. Molecules.

[13] Chen H.G., Xiao R.X., Zhou X. (2020). Study on the extraction, purification, partial chemical characterization and anti−alcohol liver injury activity of Mori Fructus polysaccharides. New J. Chem..

[14] Feng L., Yin J., Nie S., Wan Y., Xie M. (2016). Fractionation, physicochemical property and immunological activity of polysaccharides from *Cassia obtusifolia*. Int. J. Biol. Macromol..

[15] Cai L., Chen B., Yi F., Zou S. (2019). Optimization of extraction of polysaccharide from dandelion root by response surface methodology: Structural characterization and antioxidant activity. Int. J. Biol. Macromol..

[16] Long H., Gu X., Zhou N., Zhu Z., Wang C., Liu X., Zhao M. (2020). Physicochemical characterization and bile acid−binding capacity of water−extract polysaccharides fractionated by stepwise ethanol precipitation from *Caulerpa lentillifera*. Int. J. Biol. Macromol..

[17] Niu G., You G., Zhou X., Fan H., Liu X. (2023). Physicochemical properties and in vitro hypoglycemic activities of hsian−tsao polysaccharide fractions by gradient ethanol precipitation method. Int. J. Biol. Macromol..

[18] Gao W.H., Zhang P.L., Lin P.Z., Zeng X.A., Brennan M.A. (2020). Comparison of litchi polysaccharides extracted by four methods: Composition, structure and in vitro antioxidant activity. Int. J. Food Sci. Technol..

[19] Agoda−Tandjawa G., Durand S., Gaillard C., Garnier C., Doublier J. (2012). Properties of cellulose/pectins composites: Implication for structural and mechanical properties of cell wall. Carbohydr. Polym..

[20] Chen R., Li S., Liu C., Yang S., Li X. (2012). Ultrasound complex enzymes assisted extraction and biochemical activities of polysaccharides from Epimedium leaves. Process Biochem..

[21] Chen H., Zhou X., Zhang J. (2014). Optimization of enzyme assisted extraction of polysaccharides from *Astragalus membranaceus*. Carbohydr. Polym..

[22] Zhu Y., Li Q., Mao G., Zou Y., Feng W., Zheng D., Wang W., Zhou L., Zhang T., Yang J. (2014). Optimization of enzyme−assisted extraction and characterization of polysaccharides from *Hericium erinaceus*. Carbohydr. Polym..

[23] Yin X., You Q., Jiang Z. (2011). Optimization of enzyme assisted extraction of polysaccharides from Tricholoma matsutake by response surface methodology. Carbohydr. Polym..

[24] Wang S., Dong X., Tong J. (2013). Optimization of enzyme−assisted extraction of polysaccharides from alfalfa and its antioxidant activity. Int. J. Biol. Macromol..

[25] Zhang J., Jia S., Liu Y., Wu S., Ran J. (2011). Optimization of enzyme−assisted extraction of the *Lycium barbarum* polysaccharides using response surface methodology. Carbohydr. Polym..

[26] Jiao J., Fu Y.-J., Zu Y.-G., Luo M., Wang W., Zhang L., Li J. (2012). Enzyme−assisted microwave hydro−distillation essential oil from Fructus forsythia, chemical constituents, and its antimicrobial and antioxidant activities. Food Chem..

[27] Liu J., Miao S., Wen X., Sun Y. (2009). Optimization of polysaccharides (ABP) extraction from the fruiting bodies of Agaricus blazei Murill using response surface methodology (RSM). Carbohydr. Polym..

[28] Yin G., Dang Y. (2008). Optimization of extraction technology of the *Lycium barbarum* polysaccharides by Box–Behnken statistical design. Carbohydr. Polym..

[29] Xiao W., Han L., Shi B. (2008). Microwave−assisted extraction of flavonoids from Radix Astragali. Sep. Purif. Technol..

[30] Qi H., Zhao T., Zhang Q., Li Z., Zhao Z., Xing R. (2005). Antioxidant activity of different molecular weight sulfated polysaccharides from *Ulva pertusa* Kjellm (Chlorophyta). J. Appl. Phycol..

[31] Jeong J.H., Hong S.Y., Cho J.-S., Cho D.-H., Park E.Y. (2022). Impact of Enzyme Modification on Physicochemical Properties of Oat Flake and Starch. Starch−Stärke.

[32] Lv L., Cheng Y., Zheng T., Li X., Zhai R. (2014). Purification, antioxidant activity and antiglycation of polysaccharides from *Polygonum multiflorum* Thunb. Carbohydr. Polym..

[33] Chi Y., Li Y., Zhang G., Gao Y., Ye H., Gao J., Wang P. (2018). Effect of extraction techniques on properties of polysaccharides from *Enteromorpha prolifera* and their applicability in iron chelation. Carbohydr. Polym..

[34] Li X., Wang L., Wang Z. (2017). Structural characterization and antioxidant activity of polysaccharide from *Hohenbuehelia serotina*. Int. J. Biol. Macromol..

[35] Zeng D., Zhu S. (2018). Purification, characterization, antioxidant and anticancer activities of novel polysaccharides extracted from Bachu mushroom. Int. J. Biol. Macromol..

[36] Jiang F., Sheng Y., Wang F., Pan H., Chen W., Kong F. (2023). Characterization and biological activity of acidic sugarcane leaf polysaccharides by microwave−assisted hot alkali extraction. Food Biosci..

[37] Jeddou K.B., Chaari F., Maktouf S., Nouri−Ellouz O., Helbert C.B., Ghorbel R.E. (2016). Structural, functional, and antioxidant properties of water−soluble polysaccharides from potatoes peels. Food Chem..

[38] Wang D., Wang D., Yan T., Jiang W., Han X., Yan J., Guo Y. (2019). Nanostructures assembly and the property of polysaccharide extracted from Tremella Fuciformis fruiting body. Int. J. Biol. Macromol..

[39] Qin C., Huang K., Xu H. (2002). Isolation and characterization of a novel polysaccharide from the mucus of the loach, *Misgurnus anguillicaudatus*. Carbohydr. Polym..

[40] Mohammed J.K., Mahdi A.A., Ahmed M.I., Ma M., Wang H. (2020). Preparation, deproteinization, characterization, and antioxidant activity of polysaccharide from Medemia argun fruit. Int. J. Biol. Macromol..

[41] Guo R., Ai L., Cao N., Ma J., Wu Y., Wu J., Sun X. (2016). Physicochemical properties and structural characterization of a galactomannan from *Sophora alopecuroides* L. seeds. Carbohydr. Polym..

[42] Zhang Y., Xu X., Zhang L. (2008). Dynamic viscoelastic behavior of triple helical Lentinan in water: Effect of temperature. Carbohydr. Polym..

[43] Zhu J., Yu C., Han Z., Chen Z., Wei X., Wang Y. (2020). Comparative analysis of existence form for selenium and structural characteristics in artificial selenium−enriched and synthetic selenized green tea polysaccharides. Int. J. Biol. Macromol..

[44] Sun M., Li Y., Wang T., Sun Y., Xu X., Zhang Z. (2018). Isolation, fine structure and morphology studies of galactomannan from endosperm of *Gleditsia japonica* var. *delavayi*. Carbohydr. Polym..

[45] Liao X., Yang L., Chen M., Yu J., Zhang S., Ju Y. (2015). The hypoglycemic effect of a polysaccharide (GLP) from *Gracilaria lemaneiformis* and its degradation products in diabetic mice. Food Funct..

[46] Zhang Z., Wang X., Zhao M., Qi H. (2014). Free−radical degradation by Fe2+/Vc/H2O2 and antioxidant activity of polysaccharide from *Tremella fuciformis*. Carbohydr. Polym..

[47] Yuan Y., Li C., Zheng Q., Wu J., Zhu K., Shen X., Cao J. (2019). Effect of simulated gastrointestinal digestion in vitro on the antioxidant activity, molecular weight and microstructure of polysaccharides from a tropical sea cucumber (*Holothuria leucospilota*). Food Hydrocoll..

[48] DuBois M., Gilles K.A., Hamilton J.K., Rebers P.t., Smith F. (1956). Colorimetric method for determination of sugars and related substances. Anal. Chem..

[49] Bradford M. (1976). A rapid and sensitive method for the quantitation of microgram quantities of protein utilizing the principle of protein−dye binding. Anal. Biochem..

[50] Bitter T., Muir H. (1962). A modified uronic acid carbazole reaction. Anal. Biochem..

[51] Miller N.J., Rice−Evans C., Davies M.J., Gopinathan V., Milner A. (1993). A novel method for measuring antioxidant capacity and its application to monitoring the antioxidant status in premature neonates. Clin. Sci..

[52] Li X., Zhou A., Han Y. (2006). Anti−oxidation and anti−microorganism activities of purification polysaccharide from Lygodium japonicum in vitro. Carbohydr. Polym..

[53] Lin X., Ji X., Wang M., Yin S., Peng Q. (2019). An alkali−extracted polysaccharide from *Zizyphus jujuba* cv. Muzao: Structural characterizations and antioxidant activities. Int. J. Biol. Macromol..

[54] Liu W., Wang H., Pang X., Yao W., Gao X. (2010). Characterization and antioxidant activity of two low−molecular−weight polysaccharides purified from the fruiting bodies of Ganoderma lucidum. Int. J. Biol. Macromol..

